# Parthenogenetic Females of the Stick Insect *Clitarchus hookeri* Maintain Sexual Traits

**DOI:** 10.3390/insects10070202

**Published:** 2019-07-10

**Authors:** Mari Nakano, Mary Morgan-Richards, A. Jonathan R. Godfrey, Andrea Clavijo McCormick

**Affiliations:** 1 School of Agriculture and Environment, Massey University, Palmerston North 4474, New Zealand; 2 School of Fundamental Sciences, Massey University, Palmerston North 4474, New Zealand

**Keywords:** chemical signals, GC-MS, male choice, morphometrics, parthenogenetic reproduction, scramble competition, sexual reproduction, sexual traits

## Abstract

The New Zealand stick insect *Clitarchus hookeri* has both sexual and parthenogenetic (all-female) populations. Sexual populations exhibit a scramble competition mating system with distinctive sex roles, where females are signalers and males are searchers, which may lead to differences in the chemical and morphological traits between sexes. Evidence from a range of insect species has shown a decay of sexual traits is common in parthenogenetic lineages, especially those traits related to mate attraction and location, presumably due to their high cost. However, in some cases, sexual traits remain functional, either due to the recent evolution of the parthenogenetic lineage, low cost of maintenance, or because there might be an advantage in maintaining them. We measured morphological and chemical traits of *C. hookeri* to identify differences between males and females and between females from sexual and parthenogenetic populations. We also tested the ability of males to discriminate between sexual and parthenogenetic females in a laboratory bioassay. Our results show that male *C. hookeri* has morphological traits that facilitate mobility (smaller body with disproportionately longer legs) and mate detection (disproportionately longer antennae), and adult females release significantly higher amounts of volatile organic compounds than males when this species is sexually active, in accordance with their distinctive sex roles. Although some differences were detected between sexual and parthenogenetic females, the latter appear to maintain copulatory behaviors and chemical signaling. Males were unable to distinguish between sexual and parthenogenetic females, suggesting that there has been little decay in the sexual traits in the parthenogenetic lineage of *C. hookeri*.

## 1. Introduction

The dominant mode of reproduction for multicellular animals is sexual reproduction, in which male and female gametes combine through fertilization. However, some animal species (<1%) produce offspring from unfertilized eggs. This mode of reproduction is called parthenogenesis [1,2,3] and it has independently arisen in most insect groups [4]. The evidence suggests that the majority of parthenogenetic lineages evolved recently from sexual ancestors [5]. Females of some of these lineages are able to reproduce either sexually or asexually, which is known as facultative parthenogenesis [2], showing that asexual and sexual reproduction can coexist. Stick insects (Phasmidae) are ideal organisms to study this phenomenon due to their wide variety of reproductive strategies, which have led some authors to describe them as metasexual [6,7].

The New Zealand stick insect *Clitarchus hookeri* consists of both sexual and parthenogenetic populations, inferred from the sex ratio. Females from sexual populations are facultative parthenogens, i.e., they can reproduce both sexually and parthenogenetically but will predominantly reproduce sexually (we will refer to these as sexual females through the whole paper, for ease of comparison) [8]. In contrast, females from parthenogenetic populations reproduce asexually and seem to have a barrier to fertilization because, when mated with males in captivity, only ~5% of the resulting offspring were produced via sexual reproduction (these will be referred to as parthenogenetic females) [8,9]. The low heterozygosity in parthenogenetic populations of *C. hookeri* suggests automictic parthenogenesis, where meiosis occurs, but diploidy is restored [9].

Sexual populations occur mostly in the northern region of the North Island, where males and females are found in equal numbers, whereas parthenogenetic populations occur in the south-east area of the North and South Islands, where males are absent or rare [8]. The reason for this peculiar distribution is probably derived from range expansion after the Last Glacial Maximum (LGM) [8,10], because asexual reproduction is under positive selection when populations are growing, such as during range expansion [11] and when densities are low and gene flow inhibits local adaptation [12]. No ecological divergence is known to distinguish the parthenogenetic and sexual lineages, which are both silent, nocturnal, and feed on the same host plants (*Leptospermum scoparium*—mānuka and *Kunzea ericoides*—kānuka), where they also reproduce [13]. Taking advantage of this unusual system, the aim of this study was to explore morphological and chemical trait variation in *Clitarchus hookeri* to identify differences between males and females and between sexual and parthenogenetic females.

In sexual reproduction, males and females often play different roles to enhance their reproductive success. Classic roles are seen when choosing a mating partner; females are thought to be choosy while males will attempt to copulate with multiple partners due to their lower gametic and parental investment in comparison to females [14,15]. However, it is now recognized that sperm production is costly when it is produced as ejaculate and males can also become choosy when there is a high variation in female quality [14,15].

Mating systems, such as scramble competition, provide a good example of these distinctive sex roles, where males are searchers and females are signalers (in most cases) [16,17,18,19,20,21]. In scramble competition, females advertise their availability to all males who do not monopolize partners but race to locate females [22]. In this mating system, the resources (females) are often distributed equally in space; thus, individuals have equal access to mates. For the searcher (typically male), phenotypic traits that facilitate strong dispersal ability (e.g., small body with long legs) [16,17,23] and specialized sensory systems (e.g., long antennae covered with chemoreceptors) [18,20] will be favored. Olfactory cues are the most common signals used by insects, where signalers (typically females) release chemical signals that are quantitatively and/or qualitatively specific to females [16,18]. These distinctive sex roles will result in morphological and functional differences between males and females as a product of sexual selection.

Sexual populations of *Clitarchus hookeri* exhibit a scramble competition mating system, where males are searchers and females are signalers [20]. Previous evidence suggested that females use chemical signals to attract mates as males have well-developed antennae (covered with chemoreceptors) [20]. In the wild, males preferred lighter females over heavier ones, which is considered to be a preference towards younger females that are more likely to be virgins [20], indicating that they can discriminate between females of differing quality.

As mentioned earlier, *Clitarchus hookeri* has both sexual and parthenogenetic populations, which is also the case for other insect species (e.g., *Dysmicoccus brevipes* [24], *Drosophila mercatorum* [25], *Extatosoma tiaratum* [26]). These lineages are of particular interest to investigate the fate of sexual traits when asexual reproduction becomes the predominant reproductive mode. A recent review including 93 parthenogenetic animal lineages (including eight stick insect species) evidences a decay of sexual traits, especially those related to mate attraction and location, presumably due to their elevated cost, and suggests that the loss of these traits together with barriers to egg fertilization will prevent reverting back to sexual reproduction [27]. Although less common, asexual females of some species, e.g., the stick insects *Bacillus atticus* and *B. rossius*, retain functional sexual traits [27]. Furthermore, asexual hybrids of these and other stick insects in the genus *Bacillus* can revert to sexuality by shifting to androgenesis (a mode of reproduction where the male is the single source of genetic material in the embryo) during their egg development [6], suggesting that the change from sexual to asexual reproduction is not a one-way street.

Some examples of sexually-selected traits that have been reported to decay include the ability to fertilize eggs [8,28], copulatory behaviors [25,29], the sperm storage organ (i.e., spermatheca) [28,29], and chemical signals [24,26,28]. The reason for trait decay is thought to be due to traits that are non-functional becoming a fitness liability [2,27,28], but it may also be useful for asexual females to avoid unwanted mating attempts [26]. Differences in these traits can allow males to discriminate between parthenogenetic and sexual females, preferring the latter as shown in the Australian stick insect *Extatosoma tiaratum* [26].

Based on the existing literature, we predicted that morphological and chemical differences would be found between male and female *Clitarchus hookeri* due to the distinct roles of the two sexes in their scramble competition mating system. Assuming there is a cost associated with investing in sexual signaling, we predicted that we would detect a decay of chemical traits in parthenogenetic females, with a barrier to fertilization. In accordance with this prediction, we also expected male *C. hookeri* to prefer sexual females over parthenogenetic ones.

## 2. Materials and Methods

### 2.1. Sampling Location and Insect Husbandry

**Figure 1:**
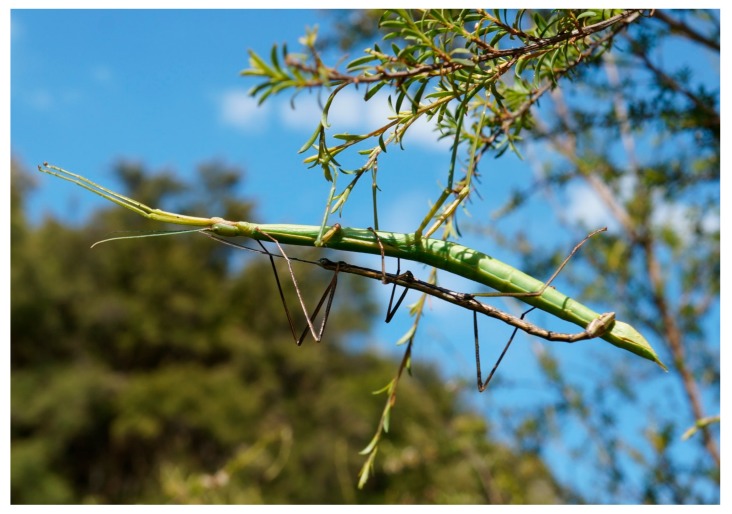
Mate guarding by the New Zealand stick insect *Clitarchus hookeri* on the host plant, kānuka, *Kunzea ericoides*. Photo credit: Steven Trewick.

**Figure 2:**
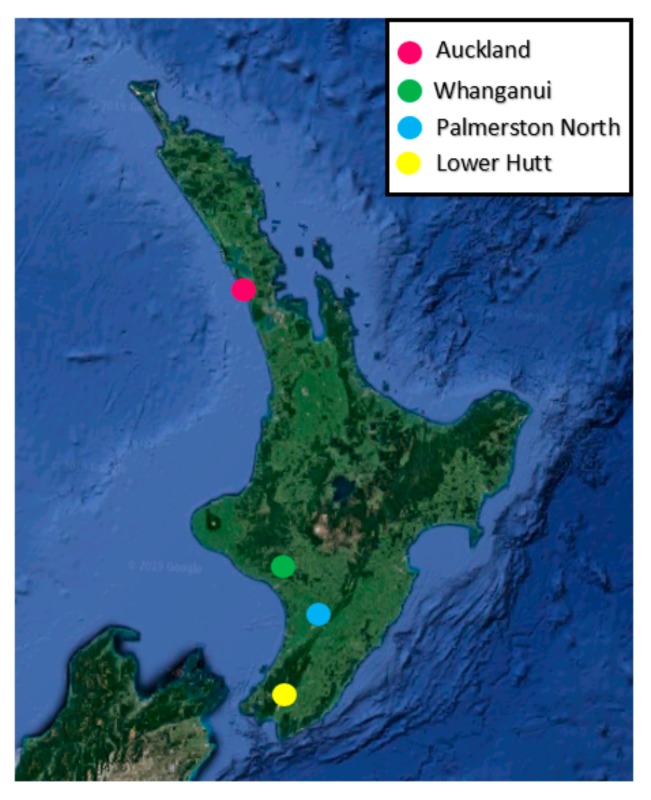
Sampling locations of *Clitarchus hookeri*, in the North Island, New Zealand.

*Clitarchus hookeri* (Figure 1) were collected from sexual and parthenogenetic populations from the North Island, New Zealand. Males and sexual females were collected from Auckland and Whanganui, where the sex ratio is 1:1 (although females are facultative parthenogens, if mated, all offspring are the result of sexual reproduction [8]). Parthenogenetic females were collected from Palmerston North and Lower Hutt, where males are absent and females have a partial barrier to fertilization (Figure 2).

Individuals were collected at the peak abundance time during the reproductive season: Auckland and Whanganui during late November to December 2017, Palmerston North during December 2017 to January 2018, and Lower Hutt during February to March 2018. Sexual females who were already adult when collected were frozen for morphological analysis.

Males, sexual females, and parthenogenetic females were kept in tanks with members of the same sex (or reproductive mode) and population. The temperature was kept at 25 °C with natural light. Branches of their food plant *Leptospermum scoparium* were provided in a plastic container filled with water, and the tanks were sprayed with water every day to prevent desiccation. Four feeding stations were provided in each tank to prevent competition. The food plants were replaced with new ones every two or three days to ensure the insects were fed with fresh plants. Tanks were cleaned weekly by removing frass and eggs and wiping with paper towels.

### 2.2. Morphological Analysis

Six morphological parameters were measured: the length of body (mm), the ratio of antennae and fore-/mid-/hind-leg length to body length (mm/mm), and the ratio of body mass to body length (g/mm) (Table 1), using electronic calipers (Q-1382, Dick Smith Electronics, Sydney, Australia) and scales (ED224S, Sartorius, Göttingen, Germany). These traits were selected as they had been reported as indicators of the sex role in scramble competition for other insect species [16,17,18].

Eleven adult individuals of each sex and mode of reproduction (i.e., males, sexual females, or parthenogenetic females) were measured from each population sample. Individuals lacking both left and right legs and/or having damaged antennae were excluded from the analyses. Live specimens were weighed every two weeks after their final molt and the maximum weight observed during the insect’s lifetime was used for the final analysis. Specimens were frozen after volatile collection and male choice experiments and the length of the body and body parts were measured after thawing.

### 2.3. Chemical Analysis

Full-body headspace collections from males and females from each population were sampled (n = 6 for each sex per population) over 10 hours (15:00–01:00), at two-hour intervals. Lights were on between 15:00–21:00, and off between 21:00–01:00 (following the New Zealand summer light period). This time frame encompasses both sexually inactive and active periods for *Clitarchus hookeri* [20]. Volatiles were collected one week after the insects’ final molt. Insect body weight was recorded before the volatile collection, and then used for calculating the quantity of volatile emissions per gram of fresh weight (ng*gFw^−1^h^−1^). All the volatile collections were made between February and April 2018 in a temperature controlled room at 25 °C.

To collect volatiles, each individual was covered with a commercial polyethylene terephthalate (PET) foil bag (40 cm), and a dynamic push-pull system (VAS, Renssealer, NY, USA) was used. During the collection, charcoal filtered air was pumped into the PET bag at 0.85L/hour and pumped out 0.80L/hour through Teflon tubes to allow a slight overpressure and prevent contamination. Volatiles were trapped with a 20 mg Hay SepQ adsorbent filter, which was placed at the tip of the Teflon tube for outgoing airflow. Sampled volatiles were eluted with 200 μL of pentane containing 10 ng/μL of nonyl acetate as an internal standard.

Samples were analyzed in a gas chromatograph mass spectrometer (GC-MS-QP2010, Shimadzu Corporation, Kyoto, Japan) using a 30 mm × 0.32 mm DB-5 column. The temperature was programmed for 3 min at 50 °C then increased to 95 °C at 5 °C/min, 145 °C at 15 °C/min, 180 °C at 10 °C/min, and finally, 200 °C at 10 °C/min. The samples were injected in split mode and the sample running time was 23.83 min in total.

Compounds were identified by comparing retention times and mass spectra to those in the NIST (National Institute of Standards and Technology) library 2005 and those compounds that had a similarity >80% were used. The quantity of each compound was calculated by comparing the peak area of the compound to that of the internal standard. For the analyses, we focused on terpenoids, being the most abundant compounds in the *Clitarchus hookeri* headspace.

### 2.4. Male Choice

Mate choice experiments (n = 42) were conducted in an open arena (a glass aquarium, H39 cm × W38 cm × D20 cm) by placing one male and two females, i.e., sexual vs. parthenogenetic females, in the same tank. Both females were from different population samples from that of the male to avoid male preference for a female from his own population. Adult females were matched by their adult molting time (so they were of a similar age) and males were chosen randomly regardless of age. Each insect was used only once to ensure that females were virgins and tests were independent. Two feeding stations (a branch of *L. scoparium* in a plastic container of water) were provided at either end of the tank, to which a female was attached, and a male was placed in the center of the tank. The room was kept at 25 °C with natural light for a circadian rhythm. To identify the reproductive mode, each female was marked on her abdomen using a colored pen (Uni Posca Permanent Marker, Mitsubishi Pencil, Japan). All insects were weighed before use as the body mass of females is probably used in male mate choice [20]. All of the male choice experiments were performed from January to March 2018.

Males were given four hours (20:00–00:00) to make a choice between the two females. During this time, the behavior of male and females was observed, including the time when males and females become active (feeding, searching), timing of pairing, and copulatory behaviors. We recorded the first choice—as first female contacted—regardless of mating.

### 2.5. Data Analysis

All the statistical analyses and figures were done using the software R studio (R version 3.6.0. Boston, MA, USA). We used a random forest algorithm to identify the most important traits distinguishing males from sexual females, and sexual from parthenogenetic females, using separate analyses for chemical and morphological traits. Random forest is a dimension reduction technique using multiple learning algorithms for feature selection and classification. A large set of decision trees are generated against a target attribute (in this case sex or mode of reproduction) and the usage statistics of each feature (trait) is then used to create a ranking. The importance of each feature relative to others is given as the mean decrease accuracy (MDA), while the out-of-bag (OOB) error estimates the prediction error of the random forest [27].

After random forest analyses, the traits having the highest MDA values for each group of traits (i.e., chemical or morphological) were further investigated using Friedman tests, followed by pairwise Mann–Whitney comparisons. These non-parametric tests were used since the datasets were not normally distributed.

A binomial chi-squared test was employed to evaluate whether the number of times *C. hookeri* males chose particular females (i.e., sexual or parthenogenetic) differed significantly from males choosing the two females in equal frequency (i.e., 0.5).

## 3. Results

### 3.1. Males vs. Females

#### 3.1.1. Morphological Analysis

**Figure 3:**
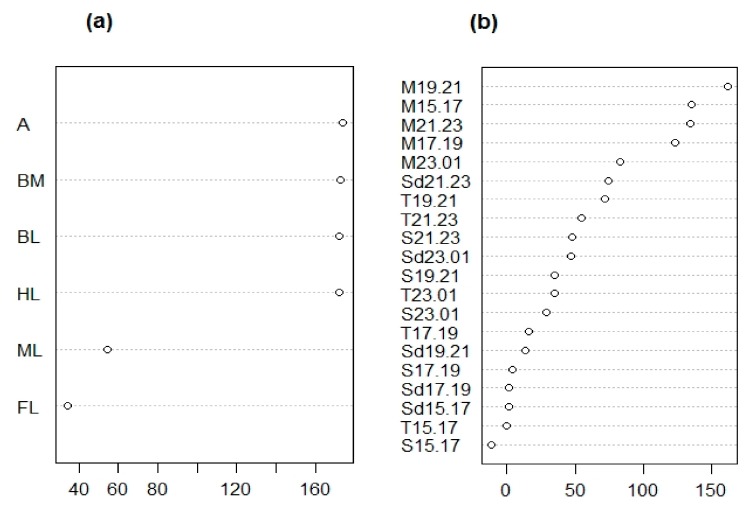
Ranking of mean decrease in accuracy (MDA) scores after a random forest analysis comparing morphological (**a**) and chemical (**b**) traits of males vs. female *Clitarchus hookeri*. (a) A statistical classification indicates the main morphological features distinguishing males and female *Clitarchus hookeri* are the antennae length ratio (A), body mass ratio (BM), total body length (BL), and hind leg length ratio (HL). (b) Chemical traits distinguishing males and females are monoterpenes (M) emitted between 15:00–23:00 in two hour intervals, i.e., 15:00–17:00 (15.17), 17:00–19:00 (17.19), 19:00–21:00 (19.21), and 21:00–23:00 (21.23). For details on the codes, see Table 1.

A random forest analysis with an out-of-bag (OOB) error of 0% indicated that it is possible to distinguish males and females based on morphological traits, and suggested several traits were equally important in this distinction (i.e., antennae length ratio, body mass ratio, total body length, and hind leg length ratio) (Figure 3a).

**Figure 4:**
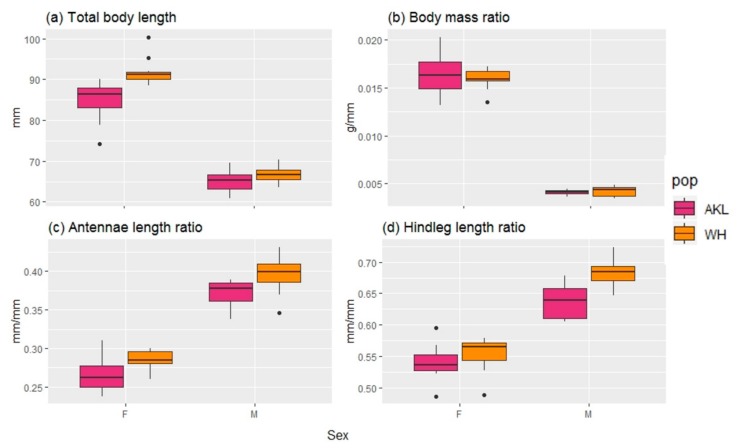
Box-and-whisker plots showing a comparison of the morphological traits of male (M) and female (F) *Clitarchus hookeri* from two sexual populations (pop) in New Zealand (AKL = Auckland, WH = Whanganui). The ends of each box represent the upper and lower quartiles, the median is marked by the vertical line inside the box. The whiskers show the highest and lowest observations, and the black points indicate outliers.

Males have shorter, disproportionately lighter bodies, and disproportionately longer antennae and hind legs compared to females (Figure 4). However, further analysis of these traits using Friedman tests, followed by pairwise comparisons, indicate that for all traits, except the body mass ratio, there are significant differences between the Auckland and Whanganui populations (Table 2). Thus, body mass is a reliable trait to distinguish males and females irrespective of their origin.

#### 3.1.2. Chemical Analysis

**Figure 5:**
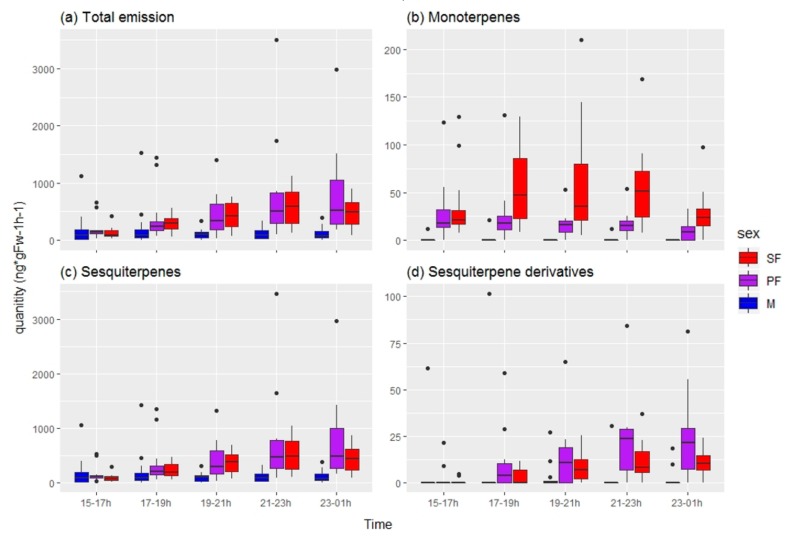
Increase in volatile emission during the night by female *Clitarchus hookeri* stick insects. SF = sexual females (red), PF = parthenogenetic females (purple), M = males (blue). Emission per individual (ng*gFw^−1^h^−1^) of (**a**) total quantity, (**b**) monoterpenes, (**c**) sesquiterpenes, and (**d**) sesquiterpene derivatives in two hour intervals. The ends of each box represent the upper and lower quartiles, the median is marked by the vertical line inside the box. The whiskers show the highest and lowest observations, and the black points indicate outliers.

In total, 24 volatile compounds were tentatively identified from *C. hookeri* by comparing their mass spectrometer (MS) profiles to those of the NIST library (Table A1). These compounds were mostly terpenoids (monoterpenes, sesquiterpenes, and sesquiterpene derivatives). We observed an increase in the total amount of volatile compounds emitted by females after 15:00 h that was significantly different to that of males (Figure 5), thus indicating that there is a sex-related difference in chemical emission, and supporting the theory of females being signalers.

Monoterpene emission between 15:00 and 23:00 h was the most important element in the distinction between males and female identified in our random forest analysis (with an OOB error of 4.17%; Figure 3b and Figure 5b). Further analyses supported the idea that that monoterpenes were responsible for distinguishing males and sexual females irrespective of population (Table 2).

### 3.2. Sexual vs. Parthenogenetic Females

#### 3.2.1. Morphological Analysis

**Figure 6:**
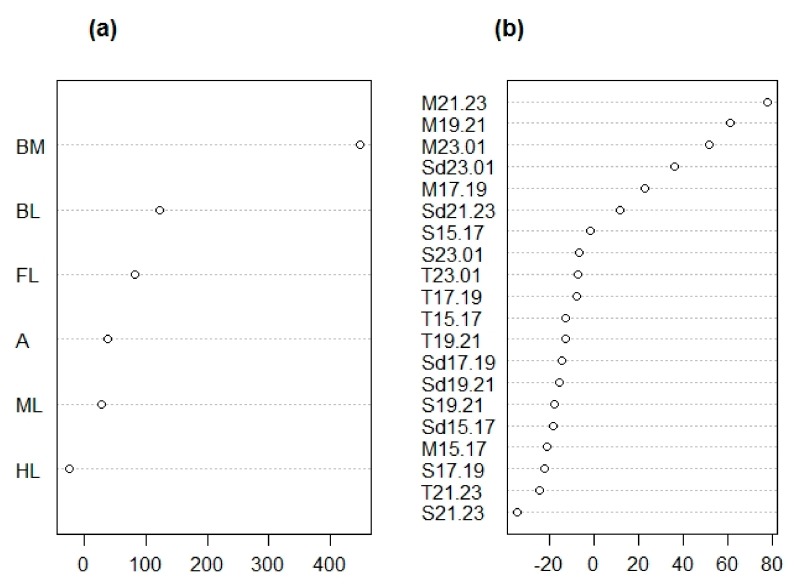
Ranking of mean decrease in accuracy (MDA) scores after a random forest analysis comparing morphological (**a**) and chemical (**b**) traits of parthenogenetic vs. sexual females of *Clitarchus hookeri*. (a) A statistical classification indicates the best morphological trait to distinguish sexual and parthenogenetic females is the body mass ratio (BM). (b) No single chemical trait was identified as distinguishing the two reproductive modes. For details on the codes, see Table 1.

**Figure 7:**
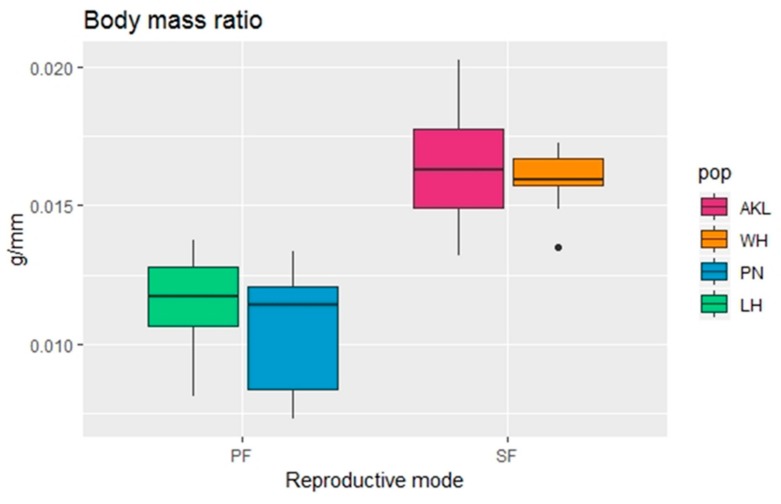
Comparison of the body mass ratio (body mass/body length; g/mm) between females of the stick insect *Clitarchus hookeri* with different reproductive modes (SF = sexual females, PF = parthenogenetic females) and populations (pop; AKL = Auckland, WH = Whanganui, PN = Palmerston North, LH = Lower Hutt). The ends of each box represent the upper and lower quartiles, the median is marked by the vertical line inside the box. The whiskers show the highest and lowest observations, and the black points indicate outliers.

A random forest analysis with an OOB error of 9.09% indicated that the body mass ratio can successfully be used to distinguish the reproductive modes of *C. hookeri* females (Figure 6a). Sexual females have a heavier body mass ratio compared to that of parthenogenetic females (Figure 7). Further analyses also indicated that the body mass ratio is responsible for differentiating sexual and parthenogenetic females without a population effect (Table 3).

#### 3.2.2. Chemical Analysis

A random forest analysis did not provide conclusive results identifying particular chemical traits as distinguishing sexual from parthenogenetic females due to the high OOB error estimated (37.5%; Figure 6b). Further analyses also failed to detect significant differences in the chemical traits between sexual and parthenogenetic *C. hookeri* females (Table 3). However, we observed an increase in the amount of monoterpenes released only by sexual females after 17:00 h (Figure 5b), suggesting a minor chemical difference may exist.

### 3.3. Male Choice

#### 3.3.1. Copulatory Behaviors

In the open arena male choice experiment, males and females were placed together at 20:00 h. Both males and females stayed inactive when it was still light; and became active in complete dark (after 21:00 h). After sunset, males started to move around the tank and/or feed on *L. scoparium*, but females were less active than males; they tended to stay on the branch and started to feed rather than moving from one branch to another. Typically, males made a choice of mating partner between 21:00 and 00:00 h. When a choice was made, the male would mount on the dorsal side of the female and become attached to the female’s operculum via his legs or clasper (modified tergite X) [20]. Males curled their abdomen to direct their poculum towards the female abdomen upon mating [20]. Rejection of males by the female involved vigorous movement (shaking their body and moving around) and the operculum staying closed even though a male was attempting copulation. If the female was stationary and her operculum opened, this was recorded as acceptance by the female. Then, the male would commence intromission by everting his phallus and inserting his genitalia into the female’s genital opening to transfer a spermatophore [20]. During intromission, the female would stay motionless or start to forage [20]. When intromission was completed, males either stayed attached to the female (i.e., mate guarding) or moved away. In the field, males were observed to mate guard females for one night on average when the adult sex ratio was 1:1 [20], which may be consistent with the duration of egg production (17 hours) in females [30].

Males from both Auckland and Whanganui were observed to mate with and mate guard sexual and parthenogenetic females, and parthenogenetic females did not reject males. Furthermore, parthenogenetic females exhibited the same copulatory behaviors observed in sexual females (i.e., opened operculum when male attempted mating and stayed either motionless or started to feed on foliage during intromission).

#### 3.3.2. Male Choice

When given a choice between a female from a sexual or parthenogenetic population, two-thirds of males chose the parthenogenetic females (28 out of 42 males), i.e., male *C. hookeri* chose parthenogenetic females significantly more than sexual females (*p* < 0.05, Chi-square test). Concurrently, males chose the lighter female significantly more often than the heavier female (27 out of 42 males; *p* < 0.05). 

## 4. Discussion

Sexual selection favors the traits that enhance access to mates [16,17,19,21]. In *Clitarchus hookeri*, males are searchers and females are signalers in their roles under scramble competition [20]. Although many morphological and chemical traits varied among populations in *C. hookeri*, males and females differ in ways that are likely to be related to their sexual roles. Males have smaller bodies (body length and body mass) with disproportionately longer legs and antennae compared to that of females. These morphological traits enhance their dispersal ability [16,17,23] and effective mate detection [18]. Conversely, females increased the amount of volatile emission at night when this species is sexually active. Terpenoid compounds (monoterpenes, sesquiterpenes, and sesquiterpene derivatives) were the most abundant group of volatiles identified in the headspace of *C. hookeri*. Terpenoids are also the major compounds that are found in their host plant, *L. scoparium* [31,32,33]. Thus, *C. hookeri* is possibly sequestering the smell from their host plants and using it dually as a camouflage mechanism and a sexual signal. Many terpenoids cannot be synthesized by plants at night due to their distinctive biosynthetic pathway that requires light for their synthesis [34,35]. Importantly, *C. hookeri* females increase their emissions of this volatile when the host plant cannot synthesize (and release) these particular chemicals, and they are doing this when their males are sexually active. This suggests that *C. hookeri* has co-opted a signal-receiving system required by the herbivore to locate food and is using it to signal and locate receptive mates [36].

Sexually selected traits can be rapidly reduced or lost entirely in parthenogenetic lineages [2,24,26,27,28]. There is evidence suggesting a decay of sexual traits in the parthenogenetic lineages of stick insects, such as *Timema* [28] and *Extatosoma tiaratum* [26]. The production of sexual signals is likely to be under negative selection in asexuals, where mate attraction is superfluous [18]. However, other stick insects (*Bacillus atticus* and *B. rossius*) retain functional sexual traits and can revert to sexuality [6], suggesting either an advantage in the maintenance of sexual traits or insufficient time for differences to develop. Models predict that parthenogenetic populations would benefit from maintaining sexual reproduction since few sporadic events of sexual reproduction are enough to maintain genetic diversity as evidenced by cyclical parthenogens, such as aphids or trematodes, which undergo several cycles of asexual reproduction followed by a sexual event [37]. A case study using the water flea, *Daphia pulicaria* (a cyclic parthenogen), supports this theory, showing that a single bout of random mating is sufficient to restore a population to Hardy–Weinberg equilibrium [38]. Reverting to sexual reproduction would only be possible when sexual traits have been maintained in parthenogenetic lineages.

In *Clitarchus hookeri*, the historical range expansion after the LGM has led to the evolution of a widespread parthenogenetic lineage, whereas sexual populations occur in all northern areas [8,10]. We initially predicted that sexual traits would be under negative selective pressure and expected to see a decay in sexual traits in the parthenogenetic lineage since these females are no longer exposed to males. However, in our analysis, only the ratio of body mass/body length differed significantly between reproductive modes, with sexual females having heavier bodies than parthenogenetic females. Large body size in females is often the result of fecundity selection [14,15,23], but in the case of *C. hookeri*, sexual and parthenogenetic females had a similar rate of egg production [8]. Thus, it is unlikely that sexual females are larger than parthenogenetic females due to stronger fecundity selection on sexual females. An alternative explanation for a larger body size in sexual females is that it may increase their ability to reject unwanted mating attempts [39] or enable them to carry males during mate guarding (which is a common behavior in stick insects [16,20,40]). If this was true for *C. hookeri*, parthenogenetic females may not be constrained to be as large as sexual females since they are not exposed to males. In some circumstances, having a small body will be a selective advantage (e.g., faster development, lower chance of being detected by predators).

None of the chemical traits were identified as being important to distinguish between sexual and parthenogenetic females. However, sexual females release higher amounts of monoterpenes than their parthenogenetic counterparts after 17:00 h. Monoterpenes emitted between 15:00 and 23:00 h were also identified as the most important chemical traits distinguishing between sexes irrespective of populations. Thus, an increase in the emission of monoterpenes may be a sex-specific sexual signal in *C. hookeri* that could eventually decay in the parthenogenetic lineage.

In some species with parthenogenetic lineages, copulatory behaviors have decayed (e.g., *Poecilimon intermedius* [41]; *Drosophila mercatorum* [25]). However, parthenogenetic *C. hookeri* females successfully copulated and did not actively reject males during our behavioral observations. Concurrently, *C. hookeri* males preferred parthenogenetic females over sexual ones. This is possibly because parthenogenetic females are on average lighter than sexual females, and male preference for lighter females was observed in a wild sexual population [20]. Although we cannot distinguish whether the reproductive mode or size of females influenced the male’s choice, we found that *C. hookeri* males did not avoid parthenogenetic females despite their barrier to fertilization. Combined, these results suggest that sexual traits (i.e., chemical signaling and copulatory behaviors) are maintained in the parthenogenetic lineage, and as a result, males failed to discriminate between sexual and parthenogenetic females.

The reason behind the maintenance of sexual traits in parthenogenetic *C. hookeri* females may be due to their particular geographical distribution. The range expansion of *C. hookeri* is likely to have occurred <40,000 years ago, which is when the parthenogenetic lineage is inferred to have evolved [9]. If correct, the relatively recent evolution of the parthenogenetic lineages in *C. hookeri* might not provide sufficient time for the loss of sexual traits. Parthenogenetic lineages may take millions of years to generate phenotypic expressed variation of sexual traits and their loss, especially when the traits evolve via drift rather than selection [27,28]. However, the barrier to fertilization detected in the parthenogenetic lineage [8] suggests rapid evolution and that sexual conflict might exist in this system, which would have implications for the long-term maintenance of both reproductive strategies.

In fact, a recent genetic study suggests that two sexual populations within the range of the parthenogenetic lineage (i.e., sexual populations in the south with mtDNA typical of parthenogenetic populations) have a mixture of sexual and parthenogenetic alleles or entirely parthenogenetic origin [9]. These recent switches in reproductive strategy suggest that although parthenogenetic females have limited capacity to reproduce sexually, it may be possible for some populations to return to sexuality. Our analyses excluded those sexual populations that have switched from parthenogenesis, but it would be revealing to know whether these populations exhibit the morphological traits that are specific to sexual or parthenogenetic reproductive modes.

## 5. Conclusions

Our study of *Clitarchus hookeri* using morphological, chemical, and behavioral analyses has shown differences between males and females in both body dimensions and chemical profiles concordant with predictions from their roles in scramble sexual competition. Both males and females smell like their host plants. However, only females increase the chemical volatiles released at night when host plants cannot synthesize these molecules and when males are searching for mates. This suggests that *Clitarchus hookeri* has co-opted a signal-receiving system required by the herbivore to locate food species (plant) and is using it to signal and locate receptive mates.

Unexpectedly, we found that the parthenogenetic lineage has maintained the female sexual signaling traits. Given the similarity of females from sexual and parthenogenetic lineages, it was not surprising that males in captivity had a preference for smaller females regardless of their reproductive mode. Although parthenogenetic females have a barrier to fertilization, selection on males to distinguish reproductive mode will be limited to regions where sexual and parthenogenetic populations naturally make contact.

Many of the morphological and chemical traits varied not only between the two sexes and reproductive modes, but among populations. Therefore, future studies should address population based analyses on morphological and chemical profiles and focus on regions where the two reproductive strategies make contact.

## Figures and Tables

**Table 1:** Morphological and chemical traits measured in this study of stick insects.

Trait ID	Trait Description	Measure	Type of Trait
BL	Total Body length	mm	Morphological
BM	Body mass ratio	g/mm	Morphological
A	Antennae length ratio	mm/mm	Morphological
FL	Front leg length ratio	mm/mm	Morphological
ML	Mid leg ratio	mm/mm	Morphological
HL	Hind leg ratio	mm/mm	Morphological
M15.17	Monoterpene emission 15–17h	ng*gFw^−1^h^−1^	Chemical
M17.19	Monoterpene emission 17–19h	ng*gFw^−1^h^−1^	Chemical
M19.21	Monoterpene emission 19–21h	ng*gFw^−1^h^−1^	Chemical
M21.23	Monoterpene emission 21–23h	ng*gFw^−1^h^−1^	Chemical
M23.01	Monoterpene emission 23–01h	ng*gFw^−1^h^−1^	Chemical
S15.17	Sesquiterpene emission 15–17h	ng*gFw^−1^h^−1^	Chemical
S17.19	Sesquiterpene emission 17–19h	ng*gFw^−1^h^−1^	Chemical
S19.21	Sesquiterpene emission 19–21h	ng*gFw^−1^h^−1^	Chemical
S21.23	Sesquiterpene emission 21–23h	ng*gFw^−1^h^−1^	Chemical
S23.01	Sesquiterpene emission 23–01h	ng*gFw^−1^h^−1^	Chemical
Sd15.17	Sesquiterpene derivative emission 15–17h	ng*gFw^−1^h^−1^	Chemical
Sd17.19	Sesquiterpene derivative emission 17–19h	ng*gFw^−1^h^−1^	Chemical
Sd19.21	Sesquiterpene derivative emission 19–21h	ng*gFw^−1^h^−1^	Chemical
Sd21.23	Sesquiterpene derivative emission 21–23h	ng*gFw^−1^h^−1^	Chemical
Sd23.01	Sesquiterpene derivative emission 23–01h	ng*gFw^−1^h^−1^	Chemical
T15.17	Total emission 15–17h	ng*gFw^−1^h^−1^	Chemical
T17.19	Total emission 17–19h	ng*gFw^−1^h^−1^	Chemical
T19.21	Total emission 19–21h	ng*gFw^−1^h^−1^	Chemical
T21.23	Total emission 21–23h	ng*gFw^−1^h^−1^	Chemical
T23.01	Total emission 23–01h	ng*gFw^−1^h^−1^	Chemical

**Table 2:** Male and female *Clitarchus hookeri* stick insects from two New Zealand sexual populations (AKL = Auckland, WH = Whanganui) differ in body shape and chemical volatiles as resolved by the Friedman test and pairwise Mann–Whitney comparisons. Total body length (BL), body mass ratio (BM), antennae length ratio (A), hind leg length ratio (HL), and monoterpenes (M) emitted between 15:00–23:00 in two hour intervals, i.e., 15:00–17:00 (15.17), 17:00–19:00 (17.19), 19:00–21:00 (19.21), and 21:00–23:00 (21.23).

Trait ID	*p*	Males		Females	
		AKL	WH	AKL	WH
BL	<0.001	a	a	b	c
BM	<0.001	a	a	b	b
A	<0.001	a	b	c	d
HL	<0.001	a	b	c	c
M15.17	<0.001	a	a	b	b
M17.19	<0.001	a	a	b	b
M19.21	<0.001	a	a	b	b
M21.23	<0.001	a	a	b	b

**Table 3:** Sexual and parthenogenetic female *Clitarchus hookeri* stick insects from four populations differ in body size but have similar chemical volatile emissions, as resolved by a Friedman test and pairwise Mann–Whitney comparisons (Sexual: AKL = Auckland, WH = Whanganui; Parthenogenetic: PN = Palmerston North, LH = Lower Hutt). Body mass ratio (BM), monoterpenes (M), and sesquiterpene derivatives (Sd), emission times 19:00–21:00 (19.21), 21:00–23:00 (21.23), and 23:00–01:00 (23.01).

Trait ID	*p*	Sexual		Parthenogenetic	
		AKL	WH	PN	LH
BM	<0.001	a	a	b	b
M19.21	0.02	a,b	a	a,b	b
M21.23	0.08				
M23.01	0.25				
Sd23.01	1				

## Appendix A

**Table A1:** List of compounds tentatively identified in the headspace samples of *Clitarchus hookeri*.

RT	Compound	Chemical Class
7.2	α-Phellandrene	Monoterpenes
7.5	α-Pinene	Monoterpenes
8.7	Sabinene	Monoterpenes
8.8	β-Pinene	Monoterpenes
9.34	β-Myrcene	Monoterpenes
11.19	α-Ocimene	Monoterpenes
11.5	γ-Terpinene	Monoterpenes
17.15	α-Cubebene	Sesquiterpenes
17.4	Ylangene	Sesquiterpenes
17.54	Copaene	Sesquiterpenes
17.63	β-Elemene	Sesquiterpenes
17.73	γ-Elemene	Sesquiterpenes
18.02	α-Gurjunene	Sesquiterpenes
18.16	β-Caryophyllene	Sesquiterpenes
18.4	Aromandrene	Sesquiterpenes
18.5	Isoledene	Sesquiterpenes
18.6	α-Caryophyllene	Sesquiterpenes
18.8	Cadina-1(10),4diene	Sesquiterpenes
18.93	GermacreneD	Sesquiterpenes
19.05	Eudesma-4(14),11-diene	Sesquiterpenes
19.12	α-Seline	Sesquiterpenes
19.24	Butylated Hydroxytoluene	Sesquiterpenes
19.43	Calamenene	Sesquiterpene derivatives
19.53	Cadine-1,4-diene	Sesquiterpenes
